# Effect of case-based learning biochemistry among first-year medical students

**DOI:** 10.6026/973206300220783

**Published:** 2026-02-28

**Authors:** Mamta Singh, Geetika Singh

**Affiliations:** 1 Department of Biochemistry, Netaji Subhas Medical College and Hospital, Bihta, Patna, Bihar, India; 2 Department of Community Medicine, Netaji Subhas Medical College and Hospital, Bihta, Patna, Bihar, India

**Keywords:** Case-based learning, biochemistry education, medical students, active learning, undergraduate medical education

## Abstract

Conventional didactic biochemistry teaching lacks clinical correlation and student engagement. Therefore, it is of interest to compare Case-Based Learning (CBL) versus traditional lectures in 85 first-year MBBS students using pre-post knowledge scores and a 10-item Likert scale perception questionnaire. CBL significantly improved median knowledge scores from 6.0 (IQR: 5.07) to 7.0 (IQR: 6.08) with high student satisfaction (weighted mean 4.23/5). Students valued CBL's interactivity, clinical relevance, critical thinking promotion and curriculum integration. CBL advances undergraduate biochemistry education as an effective, well-received interactive strategy bridging theory and practice.

## Background:

Biochemistry is another essential component of the undergraduate medical course which offers molecular and metabolic framework required to understand physiological processes, pathology and treatment [1]. Although its clinical importance cannot be underestimated, biochemistry is often viewed as a subject among medical students as an abstract, content-based subject that is difficult to memorize and use in practice due to its presentation by traditional pedagogic approaches [2]. Conventional teacher centered teaching and learning methods usually focus more in passive transfer of information and memorization which does not develop conceptual knowledge, critical thinking skills and significant clinical application among students [3]. Modern medicine education has experienced a profound shift in the paradigm of student-focused pedagogies, which emphasize active learning, partnership and the smooth incorporation of the concept of basic sciences and their application into clinical practice [4]. This educational change has had significant impetus after the introduction of competency based models of medical education worldwide, the focus of which is on the acquisition of clinical competencies and advanced cognitive skills as opposed to simple recall of facts [5]. In this changing educational environment, Case-Based Learning has become a relatively promising pedagogical approach that involves the application of authentic clinical cases to bring theoretical concepts into context thus motivating students to analyze, synthesize, discuss and apply the concepts of biochemistry in a collaborative manner [6]. Case-Based Learning is a pedagogical model where the students work with real-world clinical scenarios, which demand utilizing the basic knowledge of sciences to learn the disease pathophysiology and its treatment principles [7]. In contrast to the conventional Problem-Based Learning, CBL is often more structured with the facilitators guiding discussions and giving the required scaffolding but leaving space to the students to explore the problem by themselves and finding solutions to it together [8].

The practice has shown a significant level of effectiveness in multiple activities such as improving the higher-order cognitive abilities, such as analytical reasoning, clinical decision-making and evidence-based problem-solving, as well as increasing student motivation, engagement and satisfaction with the learning process [9]. CBL can also be a useful tool to bridge the gap between the molecular mechanisms and the clinical mechanisms of disease in such simple science fields as biochemistry, which makes the fundamental knowledge that medical students learn more applicable to medical practice in the future [10]. Studies that have been carried out in various medical education environments, have always shown positive student perception and better learning experience in the application of CBL as either an adjunct or alternative to traditional lecture-based learning [11]. The research results of various studies indicate that students who are introduced to case-based pedagogical instruction show better knowledge retention, higher analysing skills and confidence in the application of the concepts of basic science to clinical situations [12]. There has been a historical inconsistency in the application of CBL in Indian medical colleges, with few systematic assessments that assessed the application of CBL in biochemistry studies and education [13]. Since, the concept of competency based medical education and the merged concept of teaching is currently a nation-wide focus in India, there is present urge to come up with institution-specific evidence about the efficacy of new instructional methods [14]. Awareness of the perception of students and objective evaluation of learning outcomes can be a good source of advice to curriculum planners and medical educators in the efficient use of teaching-learning strategies to attain the targeted learning competencies. Moreover, the shift of competency-based curricula requires the implementation of the teaching practices that can cultivate self-direction learning, clinical thinking and practical ability to apply theoretical knowledge to real-life situations [15]. CBL relates very well to these learning outcomes as it provides an interactive learning experience where students actively generate knowledge by engaging in clinically relevant problems as opposed to being passively delivered information. Therefore, it is of interest to assess the effectiveness of Case-Based Learning versus didactic lectures in enhancing knowledge acquisition and determining the perceptions of first-year medical students of the usefulness and acceptability of CBL in undergraduate biochemistry education.

## Materials and Methods:

## Study design and setting:

In the study of assessing the effectiveness of Case-Based Learning as an undergraduate biochemistry teaching context, a quasi- experimental pre-test and post-test study design was utilized. The research was done at the Department of Biochemistry of a tertiary care teaching hospital and a medical college in Bihar, India, in the academic year 202324. The study was started with approval of the Institutional Clinical Ethics Committee. The research protocol followed all the principles of the ethical concerns of voluntary participation, informed consent, confidentiality of the response and academic integrity. They were all made aware of the study goals and were assured of the fact that their participation would not affect their academic scores.

## Participants and sample size study:

Every student who is registered in Phase I MBBS programs in the 2023-2024 academic years was eligible to participate in this study. Universal sampling was used where all the students who fit in the inclusion criteria and had given informed consent were incorporated. Eighty-five students agreed to take part and were incorporated in the analysis. The sample size was found to be sufficient in terms of prior similar researches and the limited number of the individuals accessible during the study.

## Inclusion and Exclusion Criteria:

The inclusion criteria included all first-year MBBS students who were enrolled in the 2023 2024 academic batch and attended the didactic lecture and the post didactic CBL session and gave written informed consent to participate. The students who were absent during the day of the intervention, students who failed to complete the pre-test or post-test assessment and students who refused to participate in the study were considered out of the study.

## Teaching-learning intervention:

The learning support program involved 3 consecutive segments that were implemented in one academic day to reduce the number of confounding factors in time:

## Didactic lecture:

A didactic interactive lecture, one hour long, was provided by a senior faculty covering the structure and function of the plasma membrane, different membrane transport systems (passive diffusion, facilitated diffusion, active transport and vesicular transport) and related metabolic and genetic diseases of cystic fibrosis, hereditary spherocytosis and glucose transporter deficiencies. The lecture had the use of audiovisual presentation and sought to equip the foundation of theoretical knowledge required to discuss the case later.

## Pre-test assessment:

At the very end of the didactic lecture, a pre-test test of 10 structured multiple-choice questions aiming at testing comprehension, application and recall of the main biochemical concepts taught during the didactic lecture was given to the students. There were one mark questions and a total of ten marks were allowed. The questions were formulated in accordance with the taxonomy developed by Bloom to incorporate questions that test the areas of knowledge, comprehension and application. The assessment was given to the students in 15 minutes of time.

## Case-based learning session:

After the pre-test, the students were split into small groups (8-10 members) each. Grouped case scenarios were given to the groups involving structured clinical cases on plasma membrane disorders, including the cases of clinical expression of cystic fibrosis and hereditary spherocytosis. Students have been asked to critically evaluate the clinical presentation, recognize any underlying biochemical abnormality, comment on the appropriate molecular processes and draw up evidence-based conclusions. Facilitators, who were trained, led discussions by using probing questions and by clarifying concepts where needed and active participation of all the group members. The CBL workshop took about 90 minutes.

## Post-test assessment:

At the end of the CBL session, the same MCQ questionnaire was used as a post-test with an aim of determining how knowledge improved after the case-based intervention. Cases and conditions were tested under similar conditions and time allocation was made. The participants were asked to respond to a testable 10-item feedback questionnaire, which relied on the five-point Likert scale (1 = strongly disagree, 2 = disagree, 3 = neutral, 4 = agree, 5 = strongly agree). The questionnaire evaluated various areas such as perceived usefulness in learning the subject, interactivity of the lesson, improvement of clinical association, encouragement of critical thinking, improvement of interest in biochemistry, encouragement of self-directed learning, time saving, retention of content, meaningful learning and a general recommendation on integrating the curriculum.

## Statistical analysis:

The data obtained was processed in Microsoft Excel 2019 spreadsheets and analyzed later with SPSS software version 26.0 (IBM Corporation, Armonk, NY). The continuous data distribution was tested to be normal by the use of the Kolmogorov-Smirnov test. The non- parametric statistical techniques were applied due to non-normal distribution as evidenced by the test scores. Pre-test and post-test scores were calculated as descriptive statistics (median and interquartile ranges or IQR). Wilcoxon signed-rank test was applied to compare pre-test and post-test scores that were paired. Descriptive statistics such as standard deviation, percentage distributions and mean were applied to analyze Likert scale responses. All analyses were done with a p-value at a two tailed level below 0.05 as a statistically significant level.

## Results:

A total of 85 first-year MBBS students participated in this study. All enrolled participants successfully completed the pre-test assessment, attended the complete CBL session, completed the post-test assessment and submitted the feedback questionnaire, resulting in a 100% completion rate with no missing data. There was a statistically significant improvement in knowledge scores following the Case- Based Learning intervention. The median pre-test score was 6.0 (IQR: 5.0-7.0), which increased to 7.0 (IQR: 6.0-8.0) in the post-test. This represents a median improvement of 1.0 point (16.7% relative improvement). The Wilcoxon signed-rank test demonstrated a highly significant difference between pre-test and post-test scores (Z = -4.36, p < 0.001), confirming that the observed improvement was unlikely attributable to chance (Table 1). Analysis of score distribution revealed that 71 students (83.5%) demonstrated improvement in their post-test scores compared to pre-test performance, 9 students (10.6%) maintained identical scores and only 5 students (5.9%) showed decreased scores. The maximum observed improvement was 4 points, while the minimum pre-test score was 3 and the maximum post-test score achieved was 10 (Table 1). Overall, students expressed highly positive perceptions toward the Case-Based Learning methodology. The weighted average score across all perception domains was 4.23 out of 5.0, indicating strong agreement with the benefits of CBL. The highest mean scores were observed for clinical correlation (4.51 ± 0.57), critical thinking enhancement (4.47 ± 0.63) and usefulness in understanding the topic (4.41 ± 0.54). The domain of time efficiency received the relatively lowest score (3.89 ± 0.82), although this still represented a favourable perception overall (Table 2). Analysis of categorical responses demonstrated overwhelming positive feedback regarding the CBL methodology. More than 90% of students agreed or strongly agreed that CBL promoted meaningful learning, improved content retention and facilitated self-directed learning. A substantial majority (95.3%) recommended integration of CBL into the regular biochemistry curriculum (Table 3). Regarding specific preferences, 52.9% of students strongly recommended applying CBL methodology to all biochemistry topics, while 42.4% suggested selective application to clinically relevant topics. Only 4.7% of respondents expressed reservations about expanded CBL implementation.

## Discussion:

The results of the present research can be regarded as strong evidence that Case-Based Learning is an effective method of instruction in improving both cognitive and perceptions of learners in an undergraduate level of education in the field of biochemistry. The statistically significant difference in post-test scores after CBL exposure means that there is better understanding, application and retention of biochemical concepts after exposure to didactic instruction only. The given increase in knowledge scores is consistent with the existing educational theories that focus on active learning, during which the significant interaction on the real clinical scenarios leads to the increased cognitive processing and synthesis of the theoretical knowledge [16]. Case- Based Learning promotes the idea of placing abstract biochemical pathways into concrete disease models and thus reinforcing the usefulness and importance of the underlying scientific principles to clinical practice [17]. Much of this contextualization seems to be especially useful in biochemistry, in which students usually find it difficult to see the clinical relevance of molecular mechanisms when presented using conventional didactic methods. The student perceptions of CBL are positive as noted in this research and the results of the previous studies substantiate the observation that perceiving learners as having an active participation in collaborative problem-solving tasks, as well as, being meaningfully engaged, results in greater motivation, active involvement and interest in the process [18]. Likewise, Sanghani et al. found CBL superior to interactive didactic lectures in clinical biochemistry among phase-I MBBS students, with notable gains in knowledge assessment and favourable perceptions from both students and faculty [19]. The facts that the perception scores were high in specifically enhancing critical thinking and clinical correlation in particular is indicative of the ability of well-planned CBL sessions in developing higher-order cognitive ability that is needed in making clinical decisions and providing evidence-based clinical care. These results confirm the theoretical assumption that learning is best realized when done within real-life situations that replicate real-life applications.

The strong support of CBL towards curriculum integration that has been noted in this study indicates that students can see the educational importance of this methodology even though it involves extra time and effort. Even though the idea of CBL being more time- consuming than the traditional lectures was seen as quite alarming by about one-third of participants, the attitude was overwhelmingly overshadowed by an idea of the perceived educational value, indicating that the extra time investing is being rewarded by significantly better learning quality and more substantive conceptualization [9]. Surprisingly enough, the area of clinical correlation was ranked as the highest perception score, meaning that first-year medical students, in particular, appreciate the possibility of relating the basic science with clinical practice at the beginning of their medical studies. The implications of this finding are also significant to curriculum design since this may indicate that teaching clinical relevance in preclinical teaching can increase student interest and motivation in basic sciences [4]. The findings of this paper are in line with the literature published in the basic medical sciences, as well as the clinical medical sciences that all reiterate the importance of case-based pedagogical strategies in the process of building lifelong learning skills, encouraging self-directed learning and sealing post clinical-clinical gaps in education [1]. Studies in different fields of healthcare have revealed that, students who have been exposed to case-based teaching exhibit better retention of factual knowledge, better knowledge in integrating concepts into new circumstances and better confidence in clinical reasoning [2]. It seems that the success of the CBL implementation depends on multiple factors that are organized facilitation, indication of proper correspondence to the specified learning goals, the correct level of case complexity and sufficient time allocation to meaningful discussion [3]. The trained facilitators and planned clinical cases associated with plasma membrane disorders used in this study might have been one of the contributing factors to positive outcomes achieved. Such facilitators offered the right scaffolding as well as allowing enough student-directed exploration and collaborative learning. Pedagogically, CBL seems especially appropriate in biochemistry education since it deals with one of the key issues facing students and that is the inability to see the clinical applicability of molecular and metabolic concepts [4]. CBL enables students to realize the importance of background knowledge by showing them biochemical abnormalities in the context of clinical syndromes that the students can recognize in their future as physicians. The implications of the findings as well are related to the application of competency-based medical education programs, the focus of which is on the development of integrated competencies instead of singular pieces of factual knowledge [5]. CBL, by nature, encourages knowledge integration between disciplinary boundaries and allows development of reasoning skills on which clinical competence builds. The research has a number of methodological strengths such as applying a structured pre-test and post-test design that allows making a direct comparison, full participation without any drop out, in-depth measurement of diverse areas of perception and application in a natural educational environment. Validated measuring tools and standardized procedures are also used which increases the reliability of results. Nevertheless, there are some shortcomings that should be mentioned. The study area in a single-institution context might be restrictive in generalizing to other educational settings whose student populations and institutional resources are different. The lack of parallel control group that only received didactic instruction without later CBL prevents the capability of attributing the improvements that are observed only to the CBL intervention. Also, the learning outcomes measurement was confined to the short term evaluation of learning after the interventions, not allowing drawing conclusions about the long term level of knowledge retention. Further studies ought to investigate how the retention of knowledge by delaying post-testing can be made longer, multi-institutional studies should be done to increase its generalizability and other active techniques of learning should also be compared. Research into factors that determine how individual students react to CBL would also be useful in shaping the best implementation strategies, such as their previous academic performance and their learning style preference.

## Conclusion:

Case-Based Learning (CBL) significantly enhances knowledge acquisition, clinical reasoning and student engagement in undergraduate biochemistry, as evidenced by improved post-test scores over traditional didactic methods. Students expressed strong approval for CBL's usefulness, clinical relevance and critical thinking benefits, recognizing its value for meaningful and integrated learning despite increased time demands. Integrating structured CBL sessions within biochemistry curricula aligns with competency-based medical education goals by fostering self-directed learning and practical application of biomedical concepts.

## Figures and Tables

**Table 1:** Distribution of pre-test and post-test knowledge scores (N = 85)

**Assessment Parameter**	**Pre-Test**	**Post-Test**
Median Score	6	7
First Quartile (Q1)	5	6
Third Quartile (Q3)	7	8
Interquartile Range	2	2
Minimum Score	3	4
Maximum Score	9	10
Z-value (Wilcoxon)	-4.36	-
p-value	<0.001	-

**Table 2:** Student perception of case-based learning across different domains (N = 85)

**Perception Domain**	**Mean Score**	**Standard Deviation**
Usefulness in understanding topic	4.41	±0.54
Interactivity of session	4.2	±0.75
Clinical correlation enhancement	4.51	±0.57
Critical thinking enhancement	4.47	±0.63
Increased interest in biochemistry	4.24	±0.65
Promotion of self-directed learning	4.18	±0.71
Time efficiency of method	3.89	±0.82
Content retention improvement	4.29	±0.68
Meaningful learning experience	4.35	±0.59
Recommendation for curriculum	4.42	±0.61
Overall Weighted Average	4.23	±0.66

**Table 3:** Summary of student feedback responses (N = 85)

**Feedback Parameter**	**Agree/Strongly Agree n (%)**	**Neutral n (%)**	**Disagree/Strongly Disagree n (%)**
Improved meaningful learning	79 (92.9%)	5 (5.9%)	1 (1.2%)
Better content retention	79 (92.9%)	4 (4.7%)	2 (2.4%)
Promoted self-directed learning	77 (90.6%)	6 (7.1%)	2 (2.4%)
Enhanced clinical correlation	81 (95.3%)	3 (3.5%)	1 (1.2%)
Improved critical thinking	80 (94.1%)	4 (4.7%)	1 (1.2%)
Recommended for curriculum integration	81 (95.3%)	3 (3.5%)	1 (1.2%)
Perceived as time-consuming	28 (32.9%)	19 (22.4%)	38 (44.7%)
Superior to traditional lectures	76 (89.4%)	7 (8.2%)	2 (2.4%)
